# 5-(4-Bromo­phen­yl)-2-methyl-3-methyl­sulfinyl-1-benzofuran

**DOI:** 10.1107/S1600536809033509

**Published:** 2009-08-29

**Authors:** Hong Dae Choi, Pil Ja Seo, Byeng Wha Son, Uk Lee

**Affiliations:** a Department of Chemistry, Dongeui University, San 24 Kaya-dong Busanjin-gu, Busan 614-714, Republic of Korea; b Department of Chemistry, Pukyong National University, 599-1 Daeyeon 3-dong, Nam-gu, Busan 608-737, Republic of Korea

## Abstract

In the title compound, C_16_H_13_BrO_2_S, the O atom and the methyl group of the methyl­sulfinyl substituent lie on opposite sides of the plane of the benzofuran fragment. The 4-bromo­phenyl ring is rotated out of the benzofuran plane, making a dihedral angle of 39.23 (8)°. The crystal structure exhibits weak non-classical inter­molecular C—H⋯O hydrogen bonds and two inter­molecular C—H⋯π inter­actions.

## Related literature

For the crystal structures of similar 5-aryl-2-methyl-1-benzofuran derivatives, see: Choi *et al.* (2006**a* ▶,b* ▶). For the pharmacological activity of benzofuran compounds, see: Howlett *et al.* (1999 ▶); Twyman & Allsop (1999 ▶). For natural products with benzofuran rings, see: Akgul & Anil (2003 ▶); von Reuss & König (2004 ▶).
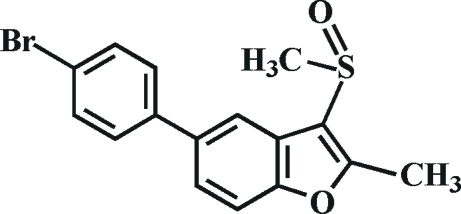


## Experimental

### 

#### Crystal data

C_16_H_13_BrO_2_S
*M* *_r_* = 349.23
Monoclinic, 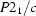

*a* = 11.410 (1) Å
*b* = 7.9508 (8) Å
*c* = 15.728 (2) Å
β = 99.399 (1)°
*V* = 1407.7 (3) Å^3^
*Z* = 4
Mo *K*α radiation
μ = 3.07 mm^−1^
*T* = 173 K
0.30 × 0.30 × 0.20 mm

#### Data collection

Bruker SMART CCD diffractometer
Absorption correction: multi-scan (*SADABS*; Sheldrick, 2000 ▶) *T*_min_ = 0.460, *T*_max_ = 0.579
8551 measured reflections
3195 independent reflections
2551 reflections with *I* > 2σ(*I*)
*R*_int_ = 0.035

#### Refinement

*R*[*F* ^2^ > 2σ(*F* ^2^)] = 0.028
*wR*(*F* ^2^) = 0.076
*S* = 1.04
3195 reflections
183 parameters
H-atom parameters constrained
Δρ_max_ = 0.52 e Å^−3^
Δρ_min_ = −0.43 e Å^−3^

### 

Data collection: *SMART* (Bruker, 2001 ▶); cell refinement: *SAINT* (Bruker, 2001 ▶); data reduction: *SAINT*; program(s) used to solve structure: *SHELXS97* (Sheldrick, 2008 ▶); program(s) used to refine structure: *SHELXL97* (Sheldrick, 2008 ▶); molecular graphics: *ORTEP-3* (Farrugia, 1997 ▶) and *DIAMOND* (Brandenburg, 1998 ▶); software used to prepare material for publication: *SHELXL97*.

## Supplementary Material

Crystal structure: contains datablocks global, I. DOI: 10.1107/S1600536809033509/zl2233sup1.cif

Structure factors: contains datablocks I. DOI: 10.1107/S1600536809033509/zl2233Isup2.hkl

Additional supplementary materials:  crystallographic information; 3D view; checkCIF report

## Figures and Tables

**Table 1:** Hydrogen-bond geometry (Å, °)

*D*—H⋯*A*	*D*—H	H⋯*A*	*D*⋯*A*	*D*—H⋯*A*
C10—H10⋯O2^i^	0.93	2.66	3.416 (3)	139
C15—H15*B*⋯O1^ii^	0.96	2.66	3.380 (3)	132
C16—H16*A*⋯O2^i^	0.96	2.63	3.463 (3)	145
C13—H13⋯*Cg*2^iii^	0.93	2.86	3.624 (3)	140
C16—H16*B*⋯*Cg*1^iv^	0.96	2.90	3.768 (3)	152

Symmetry codes: (i) 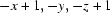; (ii) 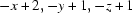; (iii) 
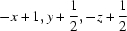
; (iv) 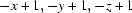. *Cg*1 and *Cg*2 are the centroids of the C9–C14 phenyl ring and the C1/C2/C7/O2/C8 furan ring, respectively.

**Fig. 1.**
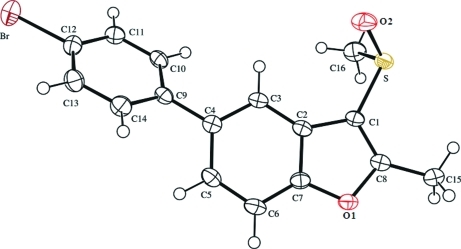
The molecular structure of the title compound with the atom numbering scheme. Displacement ellipsoids are drawn at the 50% probability level. H atoms are presented as small spheres of arbitrary radius.

**Fig. 2.**
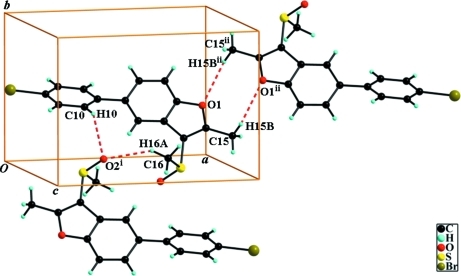
C–H···O interactions (dotted lines) in the title compound. [Symmetry codes: (i) -x+1, -y, -z+1; (ii) -x+2, -y+1, -z+1.]

**Fig. 3.**
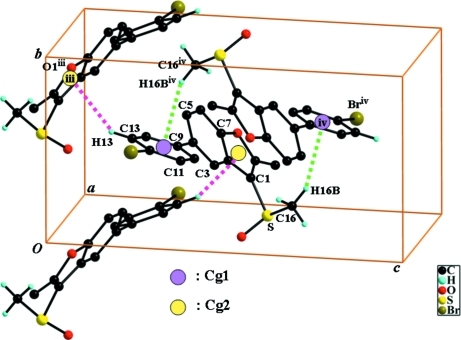
C–H···π interactions (dotted lines) in the title compound. Cg denotes the ring centroids. [Symmetry codes: (iii) -x+1, y+1/2, -z+1/2; (iv) -x + 1, -y+1, -z+1.]

## supplementary crystallographic information

### Comment

Benzofuran ring systems have attracted particular interest in the view of their pharmacological properties (Howlett *et al.*, 1999; Twyman & Allsop, 1999), and these compounds are occurring in natural products (Akgul & Anil, 2003; von Reuss & König, 2004). As a part of our ongoing studies on the synthesis and structures of 5-aryl-2-methyl-1-benzofuran analogues, the crystal structure of 5-(4-bromophenyl)-2-methyl-3-methylsulfanyl-1-benzofuran (Choi *et al.*, 2006*a*) and 2-methyl-3-methylsulfinyl-5-phenyl-1-benzofuran (Choi *et al.*, 2006*b*) have been described in the literature. Here we report the crystal structure of the title compound (Fig. 1).

The benzofuran unit is essentially planar, with a mean deviation of 0.005 (2) Å from the least-squares plane defined by the nine constituent atoms. The 4-bromophenyl ring is rotated out of the benzofuran plane, with a dihedral angle of 39.23 (8)°. The molecular packing (Fig. 2) is stabilized by weak non-classical intermolecular C–H···O hydrogen bonds; the first between the 4-bromophenyl H atom and the furan O atom, with  C10–H10···O2^i^, the second between the methyl H atom and the oxygen of the S═O unit, with C15–H15B···O1^ii^, the third between the methyl H atom of the methylsulfinyl substituent and the furan O atom, with C16–H16A···O2^i^, respectively (Table 1). The crystal packing (Fig. 3) is further stabilized by two intermolecular C–H···π interactions; the first between the 4-bromophenyl H atom and the furan ring of a neighbouring molecule, with C13–H13···Cg2^iii^, the second between the methyl H atom of the methylsulfinyl substituent and the 4-bromophenyl ring of an adjacent molecule, with C16–H16B···Cg1^iv^, respectively (Table 1; Cg1 and Cg2 are the centroids of the C9-C14 phenyl ring and the C1/C2/C7/O2/C8 furan ring, respectively).

### Experimental

77% 3-chloroperoxybenzoic acid (247 mg, 1.1 mmol) was added in small portions to a stirred solution of 5-(4-bromophenyl)-2-methyl-3-methylsulfanyl-1-benzofuran (333 mg, 1.0 mmol) in dichloromethane (40 ml) at 273 K. After being stirred for 4 hr at room temperature, the mixture was washed with saturated sodium bicarbonate solution and the organic layer was separated, dried over magnesium sulfate, filtered and concentrated in vacuum. The residue was purified by column chromatography (ethyl acetate) to afford the title compound as a colorless solid [yield 78%, m.p. 458-459 K; *R*_f_ = 0.31 (ethyl acetate)]. Single crystals suitable for X-ray diffraction were prepared by evaporation of a solution of the title compound in acetone at room temperature. Spectroscopic analysis; EI-MS 348 [M^+^], 350 [M+2].

### Refinement

All H atoms were geometrically positioned and refined using a riding model, with C–H = 0.93 Å for the aryl and 0.96 Å for the methyl H atoms. *U*_iso_(H) = 1.2*U*_eq_(C) for the aryl and 1.5*U*_eq_(C) for the methyl H atoms.

### Crystal data

**Table:**

C_16_H_13_BrO_2_S	*F*(000) = 704
*M**_r_* = 349.23	*D*_x_ = 1.648 Mg m^−^^3^
Monoclinic, *P*2_1_/*c*	Melting point = 458–459 K
Hall symbol: -P 2ybc	Mo *K*α radiation, λ = 0.71073 Å
*a* = 11.410 (1) Å	Cell parameters from 4055 reflections
*b* = 7.9508 (8) Å	θ = 2.4–27.5°
*c* = 15.728 (2) Å	µ = 3.07 mm^−^^1^
β = 99.399 (1)°	*T* = 173 K
*V* = 1407.7 (3) Å^3^	Block, colorless
*Z* = 4	0.30 × 0.30 × 0.20 mm

### Data collection

**Table:**

Bruker SMART CCD diffractometer	3195 independent reflections
Radiation source: fine-focus sealed tube	2551 reflections with *I* > 2σ(*I*)
graphite	*R*_int_ = 0.035
Detector resolution: 10.0 pixels mm^-1^	θ_max_ = 27.5°, θ_min_ = 1.8°
φ and ω scans	*h* = −14→14
Absorption correction: multi-scan (*SADABS*; Sheldrick, 2000)	*k* = −10→10
*T*_min_ = 0.460, *T*_max_ = 0.579	*l* = −13→20
8551 measured reflections	

### Refinement

**Table:**

Refinement on *F*^2^	Primary atom site location: structure-invariant direct methods
Least-squares matrix: full	Secondary atom site location: difference Fourier map
*R*[*F*^2^ > 2σ(*F*^2^)] = 0.028	Hydrogen site location: difference Fourier map
*wR*(*F*^2^) = 0.076	H-atom parameters constrained
*S* = 1.04	*w* = 1/[σ^2^(*F*_o_^2^) + (0.0363*P*)^2^ + 0.3706*P*] where *P* = (*F*_o_^2^ + 2*F*_c_^2^)/3
3195 reflections	(Δ/σ)_max_ = 0.001
183 parameters	Δρ_max_ = 0.52 e Å^−^^3^
0 restraints	Δρ_min_ = −0.43 e Å^−^^3^

### Special details

**Table:**

Geometry. All esds (except the esd in the dihedral angle between two l.s. planes) are estimated using the full covariance matrix. The cell esds are taken into account individually in the estimation of esds in distances, angles and torsion angles; correlations between esds in cell parameters are only used when they are defined by crystal symmetry. An approximate (isotropic) treatment of cell esds is used for estimating esds involving l.s. planes.
Refinement. Refinement of F^2^ against ALL reflections. The weighted R-factor wR and goodness of fit S are based on F^2^, conventional R-factors R are based on F, with F set to zero for negative F^2^. The threshold expression of F^2^ > 2sigma(F^2^) is used only for calculating R-factors(gt) etc. and is not relevant to the choice of reflections for refinement. R-factors based on F^2^ are statistically about twice as large as those based on F, and R- factors based on ALL data will be even larger.

### Fractional atomic coordinates and isotropic or equivalent isotropic displacement parameters (Å^2^)

**Table:**

	*x*	*y*	*z*	*U*_iso_*/*U*_eq_	
Br	−0.03613 (2)	0.52211 (3)	0.242047 (16)	0.03659 (10)	
S	0.73123 (5)	0.01241 (6)	0.54088 (4)	0.02489 (13)	
O1	0.86485 (12)	0.41164 (18)	0.44262 (10)	0.0257 (3)	
O2	0.64757 (15)	−0.0857 (2)	0.47714 (11)	0.0356 (4)	
C1	0.75941 (18)	0.2037 (2)	0.49091 (13)	0.0212 (4)	
C2	0.67604 (18)	0.3123 (2)	0.43719 (13)	0.0210 (4)	
C3	0.55377 (18)	0.3170 (2)	0.41169 (13)	0.0225 (4)	
H3	0.5054	0.2356	0.4306	0.027*	
C4	0.50462 (19)	0.4463 (3)	0.35708 (14)	0.0226 (4)	
C5	0.5801 (2)	0.5711 (3)	0.33141 (15)	0.0264 (5)	
H5	0.5463	0.6577	0.2959	0.032*	
C6	0.7013 (2)	0.5696 (3)	0.35685 (14)	0.0273 (5)	
H6	0.7499	0.6525	0.3397	0.033*	
C7	0.74681 (18)	0.4375 (3)	0.40949 (14)	0.0229 (4)	
C8	0.86971 (18)	0.2669 (3)	0.49163 (14)	0.0238 (4)	
C9	0.37431 (19)	0.4570 (2)	0.32810 (14)	0.0222 (4)	
C10	0.29488 (19)	0.4165 (3)	0.38373 (13)	0.0235 (4)	
H10	0.3242	0.3774	0.4388	0.028*	
C11	0.17330 (19)	0.4334 (3)	0.35854 (14)	0.0256 (5)	
H11	0.1214	0.4053	0.3962	0.031*	
C12	0.1300 (2)	0.4926 (3)	0.27679 (15)	0.0263 (5)	
C13	0.2063 (2)	0.5303 (3)	0.21940 (15)	0.0307 (5)	
H13	0.1763	0.5679	0.1641	0.037*	
C14	0.3277 (2)	0.5114 (3)	0.24500 (15)	0.0283 (5)	
H14	0.3789	0.5353	0.2063	0.034*	
C15	0.98997 (18)	0.2164 (3)	0.53466 (16)	0.0308 (5)	
H15A	0.9851	0.1114	0.5640	0.046*	
H15B	1.0215	0.3012	0.5756	0.046*	
H15C	1.0411	0.2040	0.4923	0.046*	
C16	0.6480 (2)	0.0947 (3)	0.61869 (15)	0.0338 (5)	
H16A	0.5756	0.1439	0.5895	0.051*	
H16B	0.6942	0.1790	0.6528	0.051*	
H16C	0.6296	0.0054	0.6554	0.051*	

### Atomic displacement parameters (Å^2^)

**Table:**

	*U*^11^	*U*^22^	*U*^33^	*U*^12^	*U*^13^	*U*^23^
Br	0.02728 (13)	0.04720 (17)	0.03369 (15)	0.00609 (10)	0.00023 (10)	−0.00305 (11)
S	0.0277 (3)	0.0206 (3)	0.0263 (3)	−0.0030 (2)	0.0041 (2)	0.0032 (2)
O1	0.0247 (8)	0.0238 (8)	0.0286 (8)	−0.0054 (6)	0.0047 (6)	0.0041 (6)
O2	0.0416 (10)	0.0281 (8)	0.0358 (9)	−0.0115 (7)	0.0027 (8)	−0.0036 (7)
C1	0.0264 (10)	0.0180 (10)	0.0195 (10)	−0.0035 (8)	0.0044 (8)	−0.0012 (8)
C2	0.0285 (11)	0.0174 (9)	0.0178 (10)	−0.0026 (8)	0.0062 (8)	−0.0021 (8)
C3	0.0271 (11)	0.0181 (10)	0.0231 (11)	−0.0043 (8)	0.0061 (9)	−0.0005 (8)
C4	0.0267 (11)	0.0221 (10)	0.0199 (10)	−0.0006 (8)	0.0060 (9)	−0.0022 (8)
C5	0.0327 (12)	0.0228 (10)	0.0247 (11)	0.0015 (9)	0.0079 (9)	0.0047 (9)
C6	0.0327 (12)	0.0219 (11)	0.0294 (12)	−0.0049 (9)	0.0112 (10)	0.0032 (9)
C7	0.0254 (11)	0.0216 (10)	0.0224 (11)	−0.0042 (8)	0.0059 (9)	−0.0034 (9)
C8	0.0287 (11)	0.0207 (10)	0.0226 (11)	−0.0036 (9)	0.0059 (9)	−0.0003 (8)
C9	0.0281 (11)	0.0172 (9)	0.0215 (11)	0.0001 (8)	0.0042 (9)	−0.0002 (8)
C10	0.0319 (11)	0.0208 (10)	0.0178 (10)	0.0004 (9)	0.0041 (9)	0.0023 (8)
C11	0.0289 (11)	0.0265 (11)	0.0227 (11)	−0.0022 (9)	0.0080 (9)	−0.0001 (9)
C12	0.0253 (11)	0.0266 (11)	0.0264 (12)	0.0017 (8)	0.0023 (9)	−0.0027 (9)
C13	0.0354 (12)	0.0350 (12)	0.0210 (11)	0.0005 (10)	0.0024 (10)	0.0047 (10)
C14	0.0310 (12)	0.0336 (12)	0.0216 (11)	−0.0014 (9)	0.0080 (9)	0.0035 (9)
C15	0.0259 (11)	0.0283 (12)	0.0376 (13)	−0.0052 (9)	0.0036 (10)	0.0024 (10)
C16	0.0380 (13)	0.0381 (13)	0.0268 (12)	−0.0047 (10)	0.0103 (10)	0.0019 (10)

### Geometric parameters (Å, °)

**Table:**

Br—C12	1.900 (2)	C8—C15	1.483 (3)
S—O2	1.4874 (17)	C9—C10	1.396 (3)
S—C1	1.766 (2)	C9—C14	1.397 (3)
S—C16	1.791 (2)	C10—C11	1.386 (3)
O1—C7	1.378 (3)	C10—H10	0.9300
O1—C8	1.381 (2)	C11—C12	1.382 (3)
C1—C8	1.353 (3)	C11—H11	0.9300
C1—C2	1.450 (3)	C12—C13	1.385 (3)
C2—C3	1.388 (3)	C13—C14	1.386 (3)
C2—C7	1.396 (3)	C13—H13	0.9300
C3—C4	1.397 (3)	C14—H14	0.9300
C3—H3	0.9300	C15—H15A	0.9600
C4—C5	1.415 (3)	C15—H15B	0.9600
C4—C9	1.485 (3)	C15—H15C	0.9600
C5—C6	1.376 (3)	C16—H16A	0.9600
C5—H5	0.9300	C16—H16B	0.9600
C6—C7	1.385 (3)	C16—H16C	0.9600
C6—H6	0.9300		
			
O2—S—C1	107.18 (10)	C10—C9—C4	120.96 (19)
O2—S—C16	107.39 (11)	C14—C9—C4	120.94 (19)
C1—S—C16	98.32 (11)	C11—C10—C9	121.36 (19)
C7—O1—C8	106.44 (15)	C11—C10—H10	119.3
C8—C1—C2	107.74 (17)	C9—C10—H10	119.3
C8—C1—S	123.55 (16)	C12—C11—C10	119.2 (2)
C2—C1—S	128.51 (15)	C12—C11—H11	120.4
C3—C2—C7	119.74 (19)	C10—C11—H11	120.4
C3—C2—C1	135.85 (18)	C11—C12—C13	120.8 (2)
C7—C2—C1	104.40 (17)	C11—C12—Br	119.84 (17)
C2—C3—C4	118.77 (19)	C13—C12—Br	119.31 (18)
C2—C3—H3	120.6	C12—C13—C14	119.5 (2)
C4—C3—H3	120.6	C12—C13—H13	120.3
C3—C4—C5	119.3 (2)	C14—C13—H13	120.3
C3—C4—C9	120.81 (19)	C13—C14—C9	121.0 (2)
C5—C4—C9	119.84 (19)	C13—C14—H14	119.5
C6—C5—C4	122.7 (2)	C9—C14—H14	119.5
C6—C5—H5	118.6	C8—C15—H15A	109.5
C4—C5—H5	118.6	C8—C15—H15B	109.5
C5—C6—C7	116.20 (19)	H15A—C15—H15B	109.5
C5—C6—H6	121.9	C8—C15—H15C	109.5
C7—C6—H6	121.9	H15A—C15—H15C	109.5
O1—C7—C6	125.96 (18)	H15B—C15—H15C	109.5
O1—C7—C2	110.80 (18)	S—C16—H16A	109.5
C6—C7—C2	123.2 (2)	S—C16—H16B	109.5
C1—C8—O1	110.61 (18)	H16A—C16—H16B	109.5
C1—C8—C15	133.9 (2)	S—C16—H16C	109.5
O1—C8—C15	115.47 (17)	H16A—C16—H16C	109.5
C10—C9—C14	118.1 (2)	H16B—C16—H16C	109.5
			
O2—S—C1—C8	−131.80 (18)	C1—C2—C7—C6	−179.2 (2)
C16—S—C1—C8	117.0 (2)	C2—C1—C8—O1	0.7 (2)
O2—S—C1—C2	42.4 (2)	S—C1—C8—O1	175.91 (14)
C16—S—C1—C2	−68.8 (2)	C2—C1—C8—C15	178.9 (2)
C8—C1—C2—C3	−179.4 (2)	S—C1—C8—C15	−5.9 (4)
S—C1—C2—C3	5.6 (4)	C7—O1—C8—C1	−0.7 (2)
C8—C1—C2—C7	−0.3 (2)	C7—O1—C8—C15	−179.28 (18)
S—C1—C2—C7	−175.27 (16)	C3—C4—C9—C10	37.8 (3)
C7—C2—C3—C4	1.3 (3)	C5—C4—C9—C10	−140.3 (2)
C1—C2—C3—C4	−179.7 (2)	C3—C4—C9—C14	−143.6 (2)
C2—C3—C4—C5	−1.8 (3)	C5—C4—C9—C14	38.3 (3)
C2—C3—C4—C9	−179.89 (19)	C14—C9—C10—C11	−1.6 (3)
C3—C4—C5—C6	1.1 (3)	C4—C9—C10—C11	177.03 (19)
C9—C4—C5—C6	179.2 (2)	C9—C10—C11—C12	−0.4 (3)
C4—C5—C6—C7	0.2 (3)	C10—C11—C12—C13	1.9 (3)
C8—O1—C7—C6	179.6 (2)	C10—C11—C12—Br	−178.41 (16)
C8—O1—C7—C2	0.5 (2)	C11—C12—C13—C14	−1.3 (3)
C5—C6—C7—O1	−179.8 (2)	Br—C12—C13—C14	179.01 (16)
C5—C6—C7—C2	−0.8 (3)	C12—C13—C14—C9	−0.8 (3)
C3—C2—C7—O1	179.17 (17)	C10—C9—C14—C13	2.2 (3)
C1—C2—C7—O1	−0.1 (2)	C4—C9—C14—C13	−176.4 (2)
C3—C2—C7—C6	0.1 (3)		

### Hydrogen-bond geometry (Å, °)

**Table:**

*D*—H···*A*	*D*—H	H···*A*	*D*···*A*	*D*—H···*A*
C10—H10···O2^i^	0.93	2.66	3.416 (3)	139
C15—H15B···O1^ii^	0.96	2.66	3.380 (3)	132
C16—H16A···O2^i^	0.96	2.63	3.463 (3)	145
C13—H13···Cg2^iii^	0.93	2.86	3.624 (3)	140
C16—H16B···Cg1^iv^	0.96	2.90	3.768 (3)	152

Symmetry codes: (i) −*x*+1, −*y*, −*z*+1; (ii) −*x*+2, −*y*+1, −*z*+1; (iii) −*x*+1, *y*+1/2, −*z*+1/2; (iv) −*x*+1, −*y*+1, −*z*+1.
